# Short and Long-Term Safety of the 2009 AS03-Adjuvanted Pandemic Vaccine

**DOI:** 10.1371/journal.pone.0038563

**Published:** 2012-07-03

**Authors:** Gaston De Serres, Marie-Claude Gariépy, Brenda Coleman, Isabelle Rouleau, Shelly McNeil, Mélanie Benoît, Allison McGeer, Ardith Ambrose, Judy Needham, Chantal Bergeron, Cynthia Grenier, Kenna Sleigh, Arlene Kallos, Manale Ouakki, Najwa Ouhoummane, Grant Stiver, Louis Valiquette, Anne McCarthy, Julie Bettinger, on behalf of the PHAC-CIHR influenza Research Network (PCIRN)

**Affiliations:** 1 Centre de recherche du CHUQ-CHUL, Laval University, Quebec City, Quebec, Canada; 2 Institut national de santé publique du Québec (INSPQ), Quebec City, Quebec, Canada; 3 Mount Sinai Hospital and University of Toronto, Toronto, Ontario, Canada; 4 Canadian Center for Vaccinology, Halifax, Nova Scotia, Canada; 5 Vaccine Evaluation Center, BC Children’s Hospital and the University of British Columbia, Vancouver, British Columbia, Canada; 6 The Ottawa General Hospital, Ottawa, Ontario, Canada; 7 Université de Sherbrooke, Sherbrooke, Quebec, Canada; 8 University of British Columbia, Vancouver, British Columbia, Canada; University of Hong Kong, Hong Kong

## Abstract

**Background:**

This study assessed the short and the long term safety of the 2009 AS03 adjuvanted monovalent pandemic vaccine through an active web-based electronic surveillance. We compared its safety profile to that of the seasonal trivalent inactivated influenza vaccine (TIV) for 2010–2011.

**Methodology/Principal Findings:**

Health care workers (HCW) vaccinated in 2009 with the pandemic vaccine (Arepanrix ® from GSK) or HCW vaccinated in 2010 with the 2010–2011 TIV were invited to participate in a web-based active surveillance of vaccine safety. They completed two surveys the day-8 survey covered the first 7 days post-vaccination and the day-29 survey covered events occurring 8 to 28 days after vaccination. Those who reported a problem were called by a nurse to obtain details. The main outcome was the occurrence of a new health problem or the worsening of an existing health condition that resulted in a medical consultation or work absenteeism. For the pandemic vaccine, a six-month follow-up for the occurrence of serious adverse events (SAE) was conducted. Among the 6242 HCW who received the pandemic vaccine, 440 (7%) reported 468 events compared to 328 of the 7645 HCW (4.3%) who reported 339 events after the seasonal vaccine. The 2009 pandemic vaccine was associated with significantly more local reactions than the 2010–2011 seasonal vaccine (1% vs. 0.03%, p<0.001). Paresthesia was reported by 7 HCW (0.1%) after the pandemic vaccine but by none after the seasonal vaccine. For the pandemic vaccine, no clustering of SAE was found in the 6 month follow-up.

**Conclusion:**

The 2009 pandemic vaccine seems to have a good safety profile, similar to the 2010–2011 TIV, with the exception of local reactions. This surveillance was adequately powered to identify AE associated with an excess risk ≥1 per 1000 vaccinations but is insufficient to detect rare AE.

**Trial Registration:**

ClinicalTrials.gov NCT01289418, NCT01318876

## Introduction

Influenza vaccines are generally considered safe, but the frequent changes to match the vaccine’s antigen to the influenza virus’s antigenic shifts or drifts may affect their safety profile. Unexpected adverse events have been reported with influenza vaccines including the Guillain-Barré syndrome in the United States in 1976 [1], oculorespiratory syndrome (ORS) in Canada in 2001 [2], [3] and more recently febrile convulsions that led to the suspension of the vaccination campaign in children aged 5 years and under in Australia in 2010 [4], [5]. In Canada and Europe, manufacturers are required to conduct immunogenicity trials in 60 healthy young adults and 60 elderly people to obtain their annual licensure. These trials are so small they are only able to detect adverse events occurring at a very high frequency. When the annual fall mass vaccination campaign against seasonal influenza starts, several hundreds of thousands of people are vaccinated daily over the first six weeks. At this pace, if an adverse event occurred, millions of doses may have been administered in the interval between the detection of a signal and the investigation of this AEFI. Relying on passive adverse event surveillance is insufficient and other mechanisms need to be put in place. [6] An active surveillance system capable of rapid and economical collection of safety data on a large number of vaccinated people before or very early in the mass campaign could constitute an intermediate step providing some reassurance about the safety of the vaccine.

**Table 1:** Characteristics of the health care workers vaccinated with the monovalent 2009 AS03-adjuvanted pandemic vaccine or the 2010–2011 trivalent inactivated seasonal vaccine.

	2009	2010–2011	P value
	Pandemic vaccine	Seasonal vaccine	
	n (%)	n (%)	
Participants enrolled	6525	7645	
**With valid email address**	**6242 (96%)**	**7549 (99%)**	<0.0001
Day-8 survey completed	4307(69%)	5825 (77%)	<0.0001
Day 29 survey completed	4057(65%)	5724 (76%)	<0.0001
Completed ≥1 survey	4984(80%)	6269 (83%)	<0.0001
Completed both surveys	3308(53%)	5280 (70%)	<0.0001
**Demographics**	**N = 3159** *	**N = 6280**	
Gender			0.20
Female	73%	74%	
Male	27%	26%	
Age			<0.0001
<30	33%	23%	
30–39	22%	24%	
40–49	20%	22%	
50–59	20%	23%	
60+	4%	8%	
Type of occupation			<0.0001
Physician	11%	13%	
Nurse	27%	19%	
Medical technician	6%	7%	
Other health professional	10%	16%	
Administration	11%	17%	
Other workers	34%	27%	
Vaccinated against pH1N1	100%	91%	<0.0001
Ever vaccinated against seasonal influenza	NA	91%	NA

* For 2009 this information was collected in only one site.

NA: Not available.

For its pandemic vaccination in the fall of 2009, Canada chose Arepanrix®, an AS03-adjuvanted monovalent influenza vaccine manufactured by GlaxoSmithKline (GSK). [7] Despite the experience gathered on nearly 40,000 individuals who were vaccinated in clinical trials, there were public concerns regarding the safety of the AS03 adjuvant system. [8] While passive surveillance of adverse events following immunizations (AEFI) was enhanced during the course of the mass vaccination campaign, an active electronic surveillance of a large number of health care workers (HCW) was implemented to rapidly detect unexpected adverse events associated with this vaccine. HCW were selected to participate as they were among the first individuals to receive the adjuvanted pandemic vaccine in the campaign and because they constitute a well-defined, readily accessible group highly motivated to look for AEFI. Despite the inclusion of several thousands of HCW, this cohort was designed to detect AEFI occurring at a rate ≥1 per 1000 vaccinees. This would have been sufficient for events like ORS in Canada in 2000 or seizures in Australia in 2010 but not for rare events like the Guillain Barre Syndrome (GBS). [9], [10] Many similar cohort studies have been conducted in individuals who received adjuvanted pandemic vaccines but only AEFIs occurring within the first few weeks after vaccination have been collected. [11], [12], [13], [14], [15], [16] Longer term assessment of the safety of the adjuvanted vaccine has been missing.

The objective of this study was to assess the short and the long term safety of the AS03 adjuvanted monovalent pandemic vaccine in Canada. We compared its safety profile to that of the seasonal trivalent inactivated influenza vaccine (Fluviral®, GlaxoSmithKline GSK) for 2010–2011.

## Methods

### 2.1 Study Participants and Procedures

In 2009, the surveillance was conducted in three hospitals (Quebec City, Toronto, and Halifax) whereas in 2010–2011, 7 hospitals (Quebec City, 2 in Vancouver, Toronto, Halifax, Ottawa, and Sherbrooke) participated. All HCW immunized in these institutions were invited to participate in the web-based active surveillance of vaccine safety. Participants had to sign a short consent form, provide their email address and provide their home or cell phone number. In 2009, HCW received Arepanrix®, the AS03-adjuvanted monovalent influenza vaccine from GlaxoSmithKline (GSK) whereas for 2010–2011, they were given the seasonal trivalent split inactivated influenza vaccine (Fluviral®) also from GSK.

HCW were recruited between October 26^th^ and November 12^th^, 2009 and between October 15^th^, 2010 and January 18^th^, 2011. In 2009, 8, 15 and 29 days post vaccination, participants were sent an email message requesting them to complete a standardized electronic survey accessible by clicking on a personal hyperlink embedded in the email. The day 8 survey covered the first 7 days post vaccination; the day 15 survey collected data about events occurring 8 to 14 days after vaccination; and the day 29 survey covered events occurring 15 to 28 days after vaccination. The 2010–2011 surveillance included only two surveys (one on day 8 and one on day 29); the latter covering the period from 8 to 28 days post vaccination. During both years, if a participant failed to answer within 72 hours, a reminder email was sent. Non-responders were not contacted further.

**Table 2:** New health problem or the worsening of an existing condition of sufficient significance to cause a medical consult and/or work absenteeism by follow-up period and type of influenza vaccine.

	Follow-up period										Total				
	0–7 days					8–28 days									
	Pandemic		Seasonal		RR (CI 95%)	Pandemic		Seasonal		RR (CI 95%)	Pandemic		Seasonal		RR (CI 95%)
	2009		2010–2011			2009		2010–2011			2009		2010–2011		
	n (%)		n (%)			n (%)		n (%)			n (%)		n (%)		
Number of respondents	4307	5825				4057	5724				4057	5724			
Local reaction	24 (0.56)	2 (0.03)		16.2(3.8,68.6)		15(0.37)*	0(0.0)		–		39(0.96)	2 (0.03)		27.5(6.6,113.9)	
Systemic															
Fever only	3(0.07)	30 (0.52)		0.1(0.04,0.4)		2(0.05)	19 (0.33)		0.2(0.03,0.6)		5(0.12)	49 (0.86)		0.1(0.06,0.4)	
Myalgia/fatigue with or without fever but no othersymptoms	45 (1.04)	64 (1.10)		0.9(0.6,1.4)		25(0.62)	50 (0.87)		0.7(0.4,1.1)		70(1.73)	114 (1.99)		0.9(0.6,1.2)	
Allergies	8 (0.19)	4 (0.07)		2.7(0.8,9.0)		4(0.10)	2(0.03)		2.8(0.5,15.4)		12(0.30)	6(0.10)		2.8(0.98,9.2)	
Respiratory															
Upper tract infection	86(2.00)	73 (1.25)		1.6(1.2,2.2)		99(2.44)	73 (1.28)		1.9(1.4,2.6)		185(4.56)	146 (2.55)		1.8(1.4,2.2)	
Lower tract infection	7(0.16)	6 (0.10)		1.6(0.5,4.7)		8(0.20)	11 (0.19)		1.0(0.4,2.6)		15(0.37)	17 (0.30)		1.2(0.6,2.5)	
Asthma	1(0.02)	5 (0.09)		0.3(0.03,2.3)		0	1 (0.02)		0.0(0.04,+Inf)		1(0.02)	6 (0.10)		0.2(0.03,1.9)	
Cardiac problems	3 (0.07)	1 (0.02)		4.1(0.4,39.0)		3(0.07)	2 (0.03)		2.1(0.4,12.7)		6(0.15)	3 (0.05)		2.8(0.7,11.3)	
Gastroenteritis	26(0.60)	11 (0.19)		3.2(1.6,6.5)		47(1.16)	12 (0.21)		5.5(2.9,10.4)		73(1.80)	23 (0.40)		0.2 (0.03,0.6)	
Other digestive problems	2(0.05)	6 (0.10)		0.4(0.1,2.2)		1(0.02)	5 (0.09)		0.3(0.03,2.4)		3(0.07)	11 (0.19)		0.4(0.1,1.4)	
Non-traumatic musculoskeletal problems	5(0.12)	3 (0.05)		2.3(0.5,9.4)		12(0.30)	6 (0.10)		2.8(0.98,9.2)		17(0.42)	9 (0.16)		2.7(1.2,6.0)	
Neurological															
Paresthesia/numbness	3(0.07)	0(0.0)		–		4(0.10)	0		–		7(0.17)	0		–	
Migraine	5(0.12)	3(0.05)		2.3(0.5,9.4)		5(0.12)	0		–		10(0.25)	3(0.05)		4.7(1.3,17.1)	
Urinary problems	6(0.14)	4 (0.07)		2.0(0.6,7.2)		7(0.17)	1 (0.02)		9.9(1.2,80.3)		13(0.32)	5 (0.09)		3.7(1.3,10.3)	
All others	12(0.28)	16 (0.27)		1.0(0.5,2.1)		11(0.27)	20 (0.35)		0.8(0.4,1.62)		23(0.57)	36 (0.63)		0.9(0.5,1.5)	

* These local reactions started in the 0–7 day period post vaccination but were reported in the 8–28 day survey.

The main outcome was the occurrence of a new health problem or the worsening of an existing health condition that resulted in a medical consultation or work absenteeism. For each period, participants were asked if they developed the problem during the follow-up period. Those who reported a problem on the web survey were contacted by telephone by a trained nurse who obtained a detailed history of the event. This additional information was entered within 72 hours in the central database and monitored twice weekly for potential signals by a research assistant.

For the 2009 pandemic vaccine, after the completion of the 0–28 day follow-up survey, a six-month follow-up survey for the occurrence of serious adverse events that occurred in the period from one month to six months post-pandemic vaccination was added at the request of the vaccine manufacturer. For logistical reasons, it was only conducted at the Quebec City site which included 77% of the 2009 participants. The primary outcome was the occurrence of serious adverse events (SAE) defined as any health condition requiring a hospitalization, or an event that was life-threatening, resulting in persistent or significant disability/incapacity or an event resulting in a congenital anomaly/birth defect. Participants who reported SAEs in the electronic survey were contacted by a trained nurse to obtain a detailed history regarding their reported SAE.

Each year, this study was approved by the Research Ethics Board (REB) of each participating site: Comité d’éthique de la recherche du CHUQ (Québec), Comité d’éthique de la recherche en santé chez l’humain du Centre Hospitalier universitaire de Sherbrooke (Sherbrooke), UBC C&W Research Ethics Board (Vancouver), IWK Health Centre Research Ethics Board (Halifax), Mount Sinai Hospital Research Ethics Board (REB) (Toronto), The Ottawa Hospital Research Ethics Board (Ottawa), University of British Columbia Clinical Research Ethics Board (Vancouver).

### 2.2 Analyses

AEFI were classified into broad system categories. The frequency of AEFI was compared between the 2009 pandemic vaccine and the 2010–2011 seasonal vaccine during the first 7-day post vaccine and those occurring between 8 and 28 days, using chi square test. For 2009, the results of 8–28 day follow-up were obtained by combining results from the 8–14 day survey and the 15–28 day survey. Analyses were performed using SAS version 9.2 (Inc., Cary, N.C., USA).

**Table 3:** Paresthesia of sufficient enough to cause work/school absenteeism or a medical consultation after the 2009 pandemic vaccine.

Case	Age group/Sex	Interval between vaccination and onset of symptoms	Symptoms	Interval betweenonset ofsymptoms andconsultation	Days of absenteeism
#1	35–39/Female	<1 day	Numbness to both arms accompanied by lower limbs weaknessand shoulder blade pain leading to medical consultation. Nauseaand fatigue following vaccination. Shortness of breath3–4 days post-vaccination.	3 days	2.5 days
#2	30–34/Female	1 day	Numbness to the left lower mandibula, lip and neck numbness.Tingling to the left side of the lips. Left axillary adenopathy.Headache	2 days	Not available
#3	30–34/Female	2 days	Dizziness and headache followed by tingling to the upper andlower limb extremities and abdomen. Loss of sensation andnumbness to both lower limbs.	3 weeks	Not available
#4	45–49/Female	4 days	Sudden fatigue with difficulty standing up, lower limbsnumbness, palpitation, feeling of passing out, and mild flu symptoms.Small (1 cm) local reaction 2 days post-vaccination	4 days	<1 day
#5	30–34/Male	7 days	Numbness and pain at vaccinated site. Tingling and pain tothe left side of the thorax.	11 days	None
#6	25–29/Female	12 days	Tingling from left elbow to hand including finger tips.Symptoms increased in the morning. Vaccination site tenderness12 hours post-vaccination.	Not reported	None
#7	40–44/Female	14 days	Hypoesthesia to both heels. Feet burning sensation andtingling when she showered (known past medical history).Headache	No consultation sought	Not available

## Results

A total of 6242 HCW vaccinated with the 2009 pandemic vaccine and 7549 vaccinated with the 2010–2011 seasonal influenza vaccine were enrolled and had a valid email address. (Table 1) Among them 53% and 70% respectively responded to the two surveys and 80% and 83% responded to at least one survey. In 2009, demographic information was collected at only at one site whereas all sites collected it in 2010–2011. In both years, about three quarters of participants were women. The age ranged from 16 to 87 years with about two thirds being between 30 and 60 years old while fewer than 8% were 60 years and older. The overall mean age was 39 years in 2009 and 41 years in 2010–2011. Compared to the active population in Canada, there was an over-representation of women but the mean age (40 years) was similar. [17] For both years, about 12% were physicians, 19–27% were nurses, 10–16% were other types of health professionals (pharmacists, physiotherapists, etc.) and 44%–45% were administrative or other types of workers. In 2010–2011, 91% (95% CI: 90.6, 92.0) of participants had been vaccinated against pandemic influenza the year before and 91% (95% CI: 90.0,91.5) had received seasonal influenza vaccine at least once previously.

**Table 4:** Serious adverse events (SAE) reported for the period 1–5 months after the 2009 AS03-adjuvanted monovalent pandemic vaccine.

Diagnosis		Number	Percentage
Cancer			
	Breast	1	3%
	Lung	1	3%
	Pancreas	1	3%
	Cervix (precancerous cells)	1	3%
Gynecologic/obstetrical problems			
	Ovarian cyst	1	3%
	Polymenorrhea	1	3%
	Ectopic pregnancy	1	3%
	Miscarriage (8 week pregnancy)	1	3%
	Fetal death (13 week pregnancy)	1	3%
	Hysterectomy for uterine fibroma	1	3%
Infections			
	Respiratory	3	9%
	Gastroenteritis	4	11%
	Cutaneous	2	6%
	Toxic shock syndrome	1	3%
Abdominal			
	Crohn’s disease	1	3%
	Intestinal abscess	1	3%
	Intestinal subocclusion	1	3%
	Cholecystitis	1	3%
Musculoskeletal			
	Lumbar disc hernia	1	3%
	Sprained knee	1	3%
Others			
	Depression	1	3%
	Anaphylactic shock	1	3%
	Chronic pericarditis	1	3%
	Eye/ear problem	2	6%
	Severe headache	1	3%
	Asthma	1	3%
	Vaso-vasectomy	1	3%
	No diagnosis	1	3%
	TOTAL	35	100%

Among HCW with a valid email address, for the 0–28 day follow-up, 508 (8.1%; 95% C.I: 7.5, 8.8) in 2009 and 386 (5.1%; 95% CI: 4.6, 5.6) in 2010–2011 reported on their electronic survey at least one event for which they missed work or had a medical consultation. After contact from research nurses to validate the events, 85 of the 553 events reported after the 2009 pandemic vaccine were excluded for the following reasons: 49 were erroneously reported and did not result in a medical consult or work absenteeism (main outcome) and 36 could not be validated either because the HCW did not want to divulge information about the event or the HCW was not reached. In 2010–2011, 61 of the 412 reported events were excluded after nurse follow up: 15 did not meet the criteria for the main outcome and 44 could not be validated. After these exclusions, there were 440 HCW (7% of participants; 95% CI: 6.4, 7.7) who reported 468 AEFIs after the pandemic vaccine and 328 HCW (4.3%; 95% CI: 3.9, 4.8) reported 339 AEFI after the seasonal vaccine. In both years about 80% of cases missed work for their AEFIs and about 50% consulted a physician.

As shown in Table 2, in the 28 days post vaccination, the 2009 pandemic vaccine was associated with significantly more local reactions causing work absenteeism or medical consultation than the 2010–2011 seasonal vaccine (0.96% vs. 0.03%, p<0.0001). Fever alone was significantly less frequent with the 2009 pandemic vaccine (0.12% vs. 0.86%, p<0.0001) but general malaise with or without fever was similar with both vaccines. Upper respiratory tract infection was the most commonly reported health problem during both years and was significantly more frequent with the pandemic than the seasonal vaccine (4.6% vs. 2.6%, respectively, p<0.0001). Similarly, there was more gastroenteritis reported after the pandemic vaccine than the seasonal vaccine (1.8% vs. 0.4%, p<0.0001). During the 8 to 28 day follow-up period, headache/migraine and urinary problems were reported less frequently after the 2009 pandemic vaccine than with the 2010–2011 seasonal vaccine (Table 2).

Only 3 of the 12 HCW (8 in 2009, 4 in 2010) who reported allergic symptoms 0–7 days post vaccination had their symptoms occur shortly after vaccination and all 3 had received the adjuvanted pandemic vaccine. The first case developed anaphylaxis within 30 minutes post-pandemic vaccine with generalized urticaria, swelling of the mouth and nausea. The case received adrenaline and was transferred to the emergency room. The second case developed urticaria starting 30 minutes post-vaccination followed by throat tightness an hour later. The case was transferred to the emergency room where antihistamine, corticosteroids and antacid were administered. The case was kept under observation for 4 to 5 hours and then discharged. The last case presented at the emergency department with generalized urticaria and swelling of the lips which started 5 hours post-vaccine.

In the 28 days after the 2009 pandemic vaccine, seven HCW reported paresthesia described as numbness or tingling of sufficient significance to require consultation or work absenteeism. This compared with zero reports following the 2010–2011 seasonal vaccine. (Table 3) While two HCW reported paresthesia in their vaccinated arm, four reported numbness and tingling in their lower limbs and one reported it in both upper limbs. For five of the seven HCW, symptoms started 0–7 days after vaccination.

No HCW reported diagnoses compatible with an auto-immune disease during the 28 days after receipt of the adjuvanted pandemic vaccine. In that same period, there were two SAE (hospitalization, life-threatening event, disability or stillbirth/congenital anomaly) reported for a rate of 0.32 per 1000 HCW (95% CI: 0.04,1.16) immunized. One was the first case of anaphylaxis described above. The second SAE occurred in a 55–60 year old woman with a history of diabetes and hypertension who was hospitalized for an atrioventricular block that required a pacemaker. In the 28 days after the 2010–2011 seasonal vaccine, no SAE were reported.

In the six-month follow-up after the 2009 pandemic vaccine, emails were sent to the 4,812 HCW from the Quebec City site (77% of total 2009 participants) and 3,064 (63.4%) responded. Among 68 (2.9%) participants who initially reported a SAE in the one to six month period after their pandemic vaccination, 33 were excluded after follow up by a nurse: 19 had problems that did not meet the SAE criteria, 11 had erroneously responded that they had a SAE and 3 could not be reached despite numerous attempts thus no information was available about their problem. Among the 35 HCW who met the criteria for SAE, 25 (57%) had been hospitalized, 8 (20%) reported a life-threatening event and 9 (23%) had an event resulting in persistent or significant disability/incapacity. There were no congenital anomalies/birth defects reported. Six HCW had conditions that met two criteria for a SAE with hospitalization for respiratory infections (3), hospitalization for gastrointestinal infections (4) and hospitalization for cutaneous infections (2) occurring most frequently. Otherwise the reported diagnoses affected only one patient, suggesting no cluster of SAE associated with the vaccine. (Table 4).

## Discussion

In this study, the 2009 pandemic AS03 adjuvanted vaccine used in Canada was associated with a greater frequency of AEFI in the 28 day follow-up period than the 2010–2011 unadjuvanted trivalent inactivated seasonal vaccine. While the increase in local reactions in 2009 can be attributed to the adjuvanted vaccine, the increased occurrence of upper respiratory tract infections was more likely due to the second wave of 2009 A/H1N1 which peaked at the time the HCW were vaccinated [18]. Similarly, the greater frequency of gastroenteritis seen during 2009 may have largely been attributed to an epidemic of gastroenteritis occurring at the time of vaccination in Quebec City which had 77% of the participants in 2009. The six month post pandemic vaccination follow up did not find clusters of SAE that would have been a signal for concern.

Both clinical trials and active surveillance studies have shown that pandemic adjuvanted vaccines induce frequent local reactions [11], [12], [13], [14], [15], [16], [19], [20]. Contrary to most other studies where participants were asked if they had a local reaction and how large it was, our study selected an outcome with a more direct clinical significance. HCW were asked to report AEFIs with sufficient clinical significance to seek medical advice or of enough severity to cause absence from work. There may have been some over-reporting in 2009 due to the media coverage and public concern over the pandemic, but these endpoints were likely to have minimized this problem. With the adjuvanted vaccine, local reactions were nearly thirty times more likely to trigger this outcome than the seasonal vaccine(0.96% vs. 0.03%, p<0.001). When the MF-59 adjuvanted pandemic vaccine was administered to elderly patients, local reactions were also frequently seen and 0.36% of elderly vaccinees consulted a general practitioner for this reason [13]. This higher frequency is not unexpected as both AS03 and MF59 adjuvants are designed to increase local release of chemokines to boost the immune response [21], [22]. Local reactions are not life-threatening and during a pandemic, where rapid production of a large number of doses of antigen-sparing vaccines is critical, this should not be a reason to avoid using adjuvanted vaccine. However, serious local reactions do leave a long lasting unpleasant memory and could cause reduced uptake of the vaccine in subsequent years.

Two patients had allergic symptoms within 30 minutes of their pandemic vaccine compared to none with the seasonal vaccine. The first patient met the Brighton Collaboration criteria for anaphylaxis [23]. The second had urticaria and throat tightness within 30 minutes of vaccination suggesting involvement of two systems as required for a diagnosis of anaphylaxis but in the absence of objective evidence of throat swelling it does not meet the Brighton Collaboration criteria for anaphylaxis. With only one case of anaphylaxis occurring in almost 6,000 vaccinated HCW our rate was 167 per million doses (95% CI: 4.2, 928). A review of anaphylaxis associated with Pandemrix® or Arepanrix® concluded that fewer than 100 of the worldwide reported cases in the GSK safety database met the criteria for anaphylaxis and that the rate was within the expected range of 1–10 per million doses [24]. Although our point estimate was higher, our 95% confidence interval overlapped the expected range. The passive surveillance of AEFI in Quebec received 20 times more anaphylaxis reports after Arepanrix than after the seasonal influenza vaccine for the previous 6 years [25], [26]. Using only passive surveillance data for all of Canada, 135 cases of anaphylaxis that met the Brighton Collaboration criteria were reported after vaccination with Arepanrix for a rate of about 9 per million doses and one lot of the vaccine was pulled from the supply chain because of a potential association with these reports [27]. The evidence indicates Canada experienced a higher rate of anaphylaxis after Arepanrix than that reported worldwide to the manufacturer.

The risk of auto-immune disease after the adjuvanted pandemic vaccine was not specifically sought in this surveillance project, but no HCW reported diagnoses compatible with these diseases within 28 days after vaccination.

Another unexpected AEFI that emerged from our study was the paresthesia reported by seven participants (about 1 in 1000 HCW) after the adjuvanted pandemic vaccine compared to none after the seasonal vaccine. Again, our results are supported by passive surveillance in Quebec, Canada, which in 2009, detected a strong signal for paresthesia with an early onset after the pandemic vaccine [26], [28]. Paresthesia is a frequent reason for consultation in neurology and we cannot rule out that this was coincidental rather than caused by the pandemic vaccine. However, paresthesia after Pandemrix also was reported to the passive surveillance systems in Sweden and in France [29], [30]. It also occurred at a frequency of 0.012% (6/49,138) (95% CI: 0.004%–0.026%) in French military personal participating in an active surveillance of Pandemrix [14]. Further investigation of this adverse event following AS03 vaccination appears warranted.

This study has several limitations. The main one is the non concomitant comparison of the pandemic and seasonal vaccines. The comparison may be biased because study populations differed by their geography, the hospitals included, by other factors that may have changed between the two years. The intense media attention during the pandemic may have caused over-reporting of AEFI, but the lower participation of HCW in 2009 (53% answered the two surveys) compared to 2010–2011 (70%) suggests that the impact was likely limited. We may hypothesize that non-respondents were HCW who were not motivated to respond because they did not experience an AEFI. However, we cannot rule out that answering an electronic survey would have been difficult or impossible for HCW who were very sick, hospitalized or dead. The information about the AEFI was reported by the HCW and was not validated with the attending health care providers. Classification of AEFIs in broad categories may also have obscured specific problems that would be more apparent with a more detailed stratification. Ideally, active surveillance for vaccine safety should include a group of comparable unvaccinated individuals to be in position to estimate the risks attributable to the vaccine, from background rates. In this study, the rate of background diseases (like respiratory infections or gastroenteritis) was not similar in 2009 and 2010 and this may affect the estimation of the greater reactogenicity of the adjuvanted pandemic compared to seasonal vaccine. In addition, public concerns and media attention about the adjuvanted pandemic vaccine may have lead to more comprehensive symptom reporting in 2009 than in 2010–2011 with the seasonal vaccine. Nevertheless, the main findings about the pandemic vaccine do not seem to have been substantially affected by these problems as we saw similar results in the passive surveillance system. Finally, an active surveillance of several thousand vaccinated individuals is unable to assess the risk of rare adverse events such as the Guillain Barre Syndrome.

In the six-month follow-up, 35 SAEs were reported after the adjuvanted pandemic vaccine. Although they occurred after vaccination, they are likely unrelated to the vaccine but due to other background etiologies in effect at the same time the vaccine was administered. The time-lapse from vaccination to VAE occurrence and the absence of a cluster of cases with a single diagnosis is reassuring but does not rule out the possibility of safety issues. Proper assessment of the association between these delayed SAEs and the vaccine is very difficult: it would require a much larger sample size and the comparison of their frequencies to their baseline rate in the community.

In conclusion, our surveillance has shown that the adjuvanted vaccine had a good safety profile, similar to that with the seasonal vaccine, with the exception of local reactions.
